# A Scoping Review Investigating the International Economic Evidence to Inform the Development of a Career Pathway for Home Support Workers

**DOI:** 10.3389/phrs.2025.1607091

**Published:** 2025-03-31

**Authors:** M. Lynch, E. Morrow

**Affiliations:** Faculty of Nursing and Midwifery, Royal College of Surgeons Ireland, Dublin, Ireland

**Keywords:** economic, training programs, health support worker, career pathway, continuous professional development

## Abstract

**Objectives:**

The aim of this scoping review is to explore the international evidence to identify the potential costs and gains of the development of a career pathway for Health Support Worker’s (HSW’s), the economic gains and benefits connected with continual professional development (CPD) and value for money.

**Methods:**

Scoping review following JBI methodology was conducted of peer-reviewed international literature using structured searches of electronic databases and grey literature (September 2013–November 2023) applying economic methodological terms to capture economic evidence and perspectives on the issues.

**Results:**

Seventeen papers were critically appraised and during the process of data extraction four key themes emerged: 1) Cost-benefits of employment and training 2) Organisational economic perspectives 3) Service economic perspectives and 4) Sector economic perspectives. This scoping review revealed a scarcity of economic evidence contributing to critical educational approaches, costs and benefits in development of career pathways for HSWs.

**Conclusion:**

Limited evidence was available on benefits of specific training programmes, and considerable gaps in the evidence to inform future investment. Recommendation is that future research should incorporate economic theory within evaluations to inform policy and practice.

## Introduction

Community-based care closer to home could provide more accessible and affordable care and reduce hospital admissions. However, to meet the growing demand for care and support in the community there is a need to increase workforce capacity by utilizing different types of public and private home support organizations and providers, recruiting more Home Support Workers (HSWs), and working in partnership with unpaid/family carers and community and voluntary networks. For the purposes of this article, “Home Support Worker” refers to an individual who is employed to provide support to a person in their private residence. Different terms are used internationally to describe this role (discussed later in this paper). “Home support” includes all forms of enabling support provided to or for an adult, who by reason of illness, frailty or disability needs such support and assistance [1]. However, there is considerable variation in the nature and amount of support that clients need and that is provided in different contexts.

Internationally home support sectors face challenges in meeting the demand for care and streamlining the delivery of care. There are also challenges in recruiting and retaining HSWs due to multiple factors that include poor pay, working conditions, occupational stigma, and lack of opportunities for career development and career progression [1, 2]. Furthermore, countries lack a framework – a “career pathway” – that sets out opportunities for initial training/induction, Continuing Professional Development (CPD), career development and career progression. Here, we define CPD as an ongoing planned learning and development process that enhances knowledge acquisition and understanding for role enhancement and development.

In the context of Ireland, where this scoping review was conducted, the Department of Health (DoH) has emphasized that there are workforce challenges across the health and social care system that necessitate a proactive approach to expanding home support services [3]. The government’s Sláintecare implementation strategy aims to reform and modernize Irish health and social care services [3]. The strategy is supported by a government investment of up to €15 billion in health spending. This ambitious action plan aims to deliver a health service that gives the public access to high-quality, affordable care when needed. Central to the strategy is the recruitment of an additional 2,400 healthcare workers through the Enhanced Community Care Programme, which will increase the provision of specialist teams for older individuals and those living with chronic diseases in the community.

An HSW can be defined as a person trained to look after service users (clients or patients), often older people, in their own homes. However, there is considerable variation in job titles and tasks associated with the role within Ireland and internationally. In general terms the HSW role focuses on helping individuals to live their lives with dignity and as independently as possible. In some countries, such as Ireland, the United Kingdom (UK), Germany and Canada the role of HSWs is expanding or evolving with a shift in focus from the provision of personal care or domestic duties to including care activities previously provided by Regulated Health Professionals (RHPs) (referred to as “delegated care”).

In Ireland and other nations, sector leaders and researchers have argued that HSWs should provide care and support at a level that is commensurate with their training [1, 2]. To ensure client safety and quality of care the core competencies of the role should be clearly articulated and standardized along with increased training and support for HSWs to improve job satisfaction and retention [1]. There is a strong case for the development of career pathways to facilitate the development of competencies through from initial training/induction, through regular updating and reassessment of competencies, to the development of higher levels of skills and capabilities in the workforce [2]. Developing a more qualified workforce through a structured career pathway could benefit HSWs and clients alike, bringing greater growth and expanding skillsets in the sector, offering structures for reward and recognition, and increasing stability for health systems. Workforce research in the health professions suggests that workplace learning is directly linked to employee job satisfaction and job performance – which is critical for safety and quality in the health sector [4].

Currently there is no clear career pathway, or associated CPD or career development framework for HSWs in Ireland. Alongside other limiting factors, this lack of a framework is hindering the effective recruitment and retention of HSWs into public and private provider organizations within the sector. A recent public consultation sought the views of organizations and individuals to help inform the development of draft regulations for Home Support Providers (HSPs) which included consideration of regulatory and commissioning issues [1]. The findings of this report indicated that the introduction of regulations for HSPs would significantly improve the quality and consistency of services, provide greater protection for service users, and provide greater guidance for all those involved in providing or receiving home support services. In addition, the findings of the report supported requirements for HSWs to have minimum educational qualifications that are attained within a set timeframe. The findings suggested that mandatory qualifications for HSWs could be a step towards the establishment of a career path with more structured pay and improved conditions such as opportunities for in-service learning (e.g., supervision, preceptorship or mentoring) and CPD [1].

Despite the need to increase the capacity and capability of the home support sector in Ireland and elsewhere, there could be considerable economic costs for the government, HSPs and HSWs. For example, the cost of implementing compliance assurance and meeting regulatory requirements, in terms of documentation and record keeping, along with the cost of education and training, or the cost of online professional development systems. The additional costs could be burdensome for all and undermine service provision. The counterargument is that the cost of staff turnover, which is a non-value-added element of an organization’s budget, could be far greater. This offset requires managers to focus on retention strategies to maximize the benefits of investing in trained and skilled HSWs. The huge and recurring expenses associated with high staff turnover mean that it is critical to find ways to improve employee job satisfaction and reduce turnover [5]. There are also potential cost savings to health systems if home support helps to keep people well and independent at home.

The aim of this paper is to examine international economic evidence on the actual or potential costs and benefits linked to the development of a career pathway for HSWs. The purpose is to guide the development of a career pathway for HSWs in Ireland, with insights transferable to other countries aiming to expand their home support workforce. The focus is on identifying evidence to inform decision-makers about where nations and organizations can invest or allocate economic resources to career development, training, and progression systems. This approach seeks to improve individual recruitment, retention, and progression, while also addressing the urgent need to expand and enhance workforce capability in alignment with population needs.

The objectives were to:

1) Identify relevant international economic evidence using a robust and replicable method.
2) Generate key themes from the data to highlight economic issues and identify any research gaps.

The investigation draws on a scoping review of the research literature, described below.

## Methods

A scoping review of the international research literature was chosen as a reliable and replicable method for investigating the international economic evidence [6]. It was conducted using the Joanna Briggs Institute (JBI) guidelines [7], which provide open access to guidance on the various stages involved, selection of data sources, systematic searches, presentation of data, analysis, and so on.

### Search Strategy

The search strategy was developed to identify evidence on economic issues associated with the development of a career pathway, such as, for example, CPD, career development and career progression. Key search terms and eligibility criteria were developed from the literature. The key terms used are shown in Supplementary File S1. The first section of the search includes synonyms and related job titles for “home care support worker” using OR to include all variations. The NOT section removes terms unrelated to home support (excluded terms), preventing irrelevant results from terms such as “dental devices home care” or “veterinary.” Career Framework/Pathway Terms: This section includes terms related to career progression, training, and development relevant to HSW roles. Finally, the last section captures various economic and evaluation-related terms, allowing for studies of the economic impacts, cost-benefit analysis, and quality of life measures associated with home support work. This comprehensive search structure was designed to yield targeted results across different studies and databases. An example search string is included in Supplementary File S1 for transparency and reproducibility.

### Sources

Structured searches of electronic databases were undertaken on the Web of Science, PubMed, MEDLINE, EMBASE, CINAHL, PsycINFO, Social Care Online, and Social Sciences Citation Index. To target the most recent and relevant literature the searches were limited to articles published in the last 10 years (January 2013 to November 2023). References and abstracts were downloaded to Microsoft Excel for screening.

In addition to the structured searches, additional desk-based searches were conducted for “gray” literature (e.g., research reports, conference proceedings), and advice was sought from experts in the field in collaboration with the commissioners of the study. These additional sources of information provided a realist perspective on “what works, for whom and under what circumstances?” [8] and informed the discussion of the findings.

### Eligibility Criteria

Inclusion and exclusion criteria were developed from the literature and applied to screen the articles. Article screening of titles, abstracts and full texts was conducted by two independent reviewers (EM, ML) to minimize bias and ensure rigor.

### Charting

Full copies of included articles were downloaded from journal websites and were stored electronically. Data were extracted and charted in tables in Microsoft Word to generate a bibliography of international economic evidence (Supplementary File S2).

### Assessment of the Level of Evidence

To gain insight into the overall level of international economic evidence, each article was assessed using the GRADE (Grading of Recommendations, Assessment, Development and Evaluation) approach [9]. Assessing the level of the available evidence is important for the development of reliable recommendations and ensuring transparency about the available evidence. Applying the GRADE criteria (further detail in Supplementary File S2), involves consideration of the following domains: the level of risk of bias; assessment of the inconsistency of the evidence, evaluation of the indirectness and effect of the evidence; imprecision of the results and publication bias. Taking these domains into consideration an overall assessment is made to categorize the evidence as high, moderate or low.

### Analysis

A thematic analysis was conducted using an inductive-deductive approach. This approach was chosen as a technique to identify key issues and themes within the data. Thematic analysis of the included studies followed the processes of familiarization to understand the focus of the articles, identification of preliminary themes during data extraction, charting, and development of a thematic code framework. Theme headings were derived from the data and themes were reviewed and validated by the authors. A narrative was developed to describe the topics and issues covered by each of the four themes.

## Results

In this scoping review, 17 studies that met the inclusion criteria, provided economic evidence. Further details of the article types, titles, topics, methods and content of each article are provided in Supplementary File S2.

Thematic analysis revealed four themes:

1) Costs and benefits of employment and training (7 articles)
2) Sectoral economic perspectives (5 articles)
3) Organizational economic perspectives (3 articles)
4) Service economics perspectives (2 articles)

Each theme is described below in order of its strength in the literature.

### Theme 1: Costs and Benefits of Employment and Training

Seven articles examined the first theme [10–16]. The level of evidence of the articles revolving around this theme was assessed as moderate (n = 6) and low (n = 1).

Research conducted in the United States by Luz et al., 2018 [10] examined the need for qualified, paid Personal Care Aides (PCAs) and the provision of adequate training focused on skills for person-centered care delivery in clients’ homes. Results indicate that clients report higher satisfaction and better health outcomes along with significantly fewer falls and emergency department visits compared to clients whose PCAs had no specialized PCA training, along with a reduction in costly adverse events. Further details of the study are provided in the table in Supplementary File S2. Supporting this evidence, a 2015 study by Luz and Hanson [11] showed that the introduction of a PCA training program significantly improved learners’ skills, employability, and job satisfaction. It also showed that PCAs’ intent to stay was associated with increased confidence in their ability to undertake the role.

In another study of training in the United States, Fong et al., 2022 [12] examined the impact of a workforce training intervention on value-based payment measures in a large home-based population in New York. Evaluation results indicate that workforce training could benefit high-need and long-term care recipients along with clients with average levels of need.

Kemeny and Mabry, 2017 [13]conducted a mixed methods research study to investigate the relationships between best practices in Direct Care Worker (DCW) training and the structure and culture of long-term support services (LTSS). The findings suggest that public policy should address methods of training, not just content, and consider organizational variations in size, training evaluation practices, DCW integration, and DCW input in care planning. In addition, effective training that also incorporates support for organizations and supervisors is an important key aspect of the learning and working environment for DCWs.

Ayalon and Shinan-Altman, 2021 evaluated a training program for paid elder care workers. They documented that three quarters of the participants completed the training course but only one quarter of the participants continued to work as care workers afterward [14]. Three main challenges were identified: first, the gap between the vision of the program and real-life requirements and constraints. The second challenge concerned the definition of the new role which promoted personal growth compared with the traditional direct paid carer. The third challenge concerned the program’s lack of integration between personal/physical care and emotional and psychological care. The findings stress the importance of conducting an adequate needs assessment before embarking on a new social program and the tension between an ideal prototype and real-life constraints. The findings also highlight the necessity for top-down processes, supported by the government to develop a new profession of community elderly care. However, this should not compromise the quality of qualifications and should include academic and practice-based learning [15].

Snyder et al., 2018 examined career transitions among individuals in selected entry-level healthcare occupations such as HSW and their findings indicate that there is limited evidence of career progression in healthcare for individuals who remain in their occupations [16]. However, the findings also suggest that employers and educational institutions should consider efforts to help clarify pathways to advance the careers of individuals in entry-level healthcare occupations.

### Theme 2: Sector Economic Perspectives

Under this theme four articles were deemed relevant [17–20] with a moderate level of evidence, along with a piece of gray literature that explored the pay gap in social care and how to close it [21].

A review of the literature exploring economic evaluation methods [17] identified discrepancies in the reporting of comparisons between outcomes with impacts on the decisions on the most appropriate healthcare interventions. Findings suggest that to be effective, the appropriate economic methodological approach should be central to the context and content of the specific interventions and conditions being evaluated.

Recent evidence exploring the costs and benefits of large-scale policy-directives or workforce development interventions evaluated the implementation of the Health Career Access Program (HCAP) for HSWs and feedback was positive from both providers and care receivers. Results also suggest that investing in and supporting the growth of an individual, reinforces the emphasis on the sustainment and longevity of a career building strategy for healthcare professionals [18].

Exploring the supply and demand challenges in the home care service market research investigated the difficulties arising from the market-oriented operation mechanism in optimizing the allocation of resources. Findings suggest that encouraging participants to diversify home-based care services, both providers and modes of provision could greatly improve the QOL (quality of life) of the elderly [19]. Research examining the cost-effectiveness and cost-utility of staff training [20] found that future research should integrate sound economic theory and that economic evaluation should include Social Cost Benefit Analysis (SCBA).

In light of the crisis in the social care workforce an investigative report looked at the pay of frontline carers compared with other publicly funded industries and challenged the categorization that social care is a “low-skilled” sector. The findings suggest that disparity and pay gap are at the heart of the crisis affecting social care. However, limited attention was given to the costs and benefits of the social care workforce, given that social care contributes £46 bn to the national economy each year, social care creates 1.65 m jobs each contributing to their local economy, and the sector loses 34% of its workforce each year [21].

### Theme 3: Organizational Economic Perspectives

Three articles focused on this theme [22–24] and the level of evidence from the articles included in this theme was assessed as moderate (n = 1) and low (n = 2) with the latter articles adopting a commentary approach to this topic.

Qualitative research examining efficiency requirements and managerial roles in the elderly care sector found that managers perceive that a new form of home care is emerging, where the main role of managers is to guide staff toward smarter use of resources demonstrating a shift in thinking [22]. However, this shift in thinking needs to take into account organizational economic views to address the organizational capacity to implement and deliver training agendas to include progression within the HSW role seen as a means of opening up the workforce. Central to this is the pre-eminent objective of HSW career development and supporting HSWs to grow within the role itself, enabling them to deliver high-quality care.

Issues of organizational capacity to deliver on the HSW agenda together with concerns about how to align the pursuit of different aims with the appropriate training and development programs. Career progression within the HSW role CPD training should be cognizant of the various roles, and training design options available, such as the higher development award while staying in their existing role [23]. To understand career progression, the perspective of HSWs needs to be considered along with the importance of functional skills, associated with limited funding for HSW training opportunities as a result of staff shortages [24].

### Theme 4: Service Economic Perspectives

Two articles focused on this theme [25, 26] and the level of evidence in both articles was assessed as moderate.

Research was conducted to examine the benefits of workforce training in long-term care. The evaluation of a feasibility study was designed to determine the effects of a communication intervention on residents’ quality of life (QOL) and care, along with care providers’ perceived knowledge, mood, and burden. Results indicate that residents experienced a substantial increase in overall QOL and care providers had significant improvement in mood and perceived reduced burden [25]. Qualitative research investigating the costs and benefits of payment models examined the roles and tasks of HSWs along with the costs of improving efficiency and quality of care. Findings suggest that aligning payment models with quality requires an understanding of the full scope of services HSWs provide and a quality work environment that offers support and supervision, engages HSWs in patient care, and provides a voice in policy decisions [26].

## Discussion

This scoping review highlights several key economic challenges in supporting the training, CPD, career development and career progression of HSWs. A major gap exists in the conduct of comprehensive economic evaluations, especially cost-benefit analyses (CBAs) of CPD. Employment and training have been shown to yield benefits, such as improved skills, job satisfaction, and cost advantages in high-dependency and long-term care. Sustainable sector funding remains challenging, underscoring the need for needs assessment, workforce planning, and program evaluation for HSW career development. Limited evidence on the economic value of qualifications and career pathways suggests a need to balance academic and practical learning.

Organizational perspectives, particularly regarding capacity and funding for training, are sparse, as is research on service economics; however, studies link workforce training to better outcomes for care recipients. Economic issues in the home support sector - pay disparities and staff shortages—are critical, demanding urgent economic solutions. Future research should adopt rigorous economic evaluation techniques, integrating cost-effectiveness, cost-utility, and Social Cost-Benefit Analysis (SCBA) in line with the Green Book guidance [27]. Investment in career pathways for HSWs is vital to both the sustainability of careers and the ability of the healthcare system to meet the growing demand for home-based and digitally supported care.

Evidence on the co-benefits of employment and training suggests that financial costs are offset by benefits such as increased service user satisfaction, improved health outcomes, and reductions in adverse events and healthcare usage [10]. The review highlights significant improvements in skills, employment opportunities, and job satisfaction, with training proving especially beneficial in high-dependency and long-term care settings [11, 12]. Strategic planning should integrate training and learning into work environments [13]. However, numerous challenges remain, and it is essential that governing organizations conduct needs assessments before developing and implementing new programs to support the progression of new roles in home support. Qualifications should encompass both academic and practice-based learning [14, 15]. Given the limited evidence on career progression, employers and educational institutions should consider ways to simplify pathways to facilitate career advancement for health support workers [16].

Research on organizational economic perspectives reveals limited available evidence; however, findings suggest that management perspectives advocate for more strategic use of resources by focusing on organizational capacity to implement and sustain training programs that support career progression within the role of HSWs [22]. Additionally, research into the views of HSWs is essential, particularly in relation to accessible CPD that considers their diverse roles, limited funding for training opportunities, and ongoing staff shortages.

This scoping review highlights the lack of research on service economics perspectives, with minimal evidence currently available. Findings suggest that investment in workforce training in long-term care may increase the quality of life (QOL) of end users, along with notable improvements in the attitudes of care providers [25]. Research into the costs and benefits of improving the efficiency and quality of care indicates that supportive and supervised work settings that actively involve HSWs in patient care, can lead to more effective health planning decisions [26]. Furthermore, evidence suggests that promoting and expanding home-based care services, through improved provider approaches and delivery methods, could significantly improve the QOL of the population [19].

There are several contextual limitations to these findings. First, the search and the studies reviewed encompass healthcare workers in a range of care and clinical settings along with HSWs. It is evident that the costs and benefits of CPD and other career development or progression strategies are likely to vary significantly between these roles and settings. For an international journal audience, it is essential to highlight that this is an international scoping review. However, this also means that the roles, regulation, training, and positioning of HSWs within their respective health and care systems will differ from country to country, with implications for both costs and benefits in different settings.

Insights into the economic perspectives of the home support sector reveal the need to examine economic evaluation approaches, workforce supply-and-demand policies, and the widening pay gap that contributes to the ongoing staffing crisis. Significant disparities and a growing pay gap are at the core of the challenges impacting social care; however, there has been limited focus on assessing the sector’s costs and benefits. Social care contributes £46 billion to the national economy each year, creating 1.65 million jobs and supporting local economies. However, the sector faces high staff turnover, with 34% of the workforce leaving each year [21].

Research applying rigorous economic evaluation techniques is extremely limited; however, the findings suggest that economic methodologies should be incorporated into all future evaluations to effectively capture inputs, outputs, outcomes, and impacts, and to demonstrate their monetary value and benefits. Recent economic evidence supports this, indicating that investment in HSW career pathways strengthens career sustainability and longevity in healthcare roles [18]. Building on this approach, by addressing the supply and demand challenges in the home care market, and improving resource allocation, findings suggest that the cost-effectiveness and cost-utility in staff training are crucial [20].

Limited evidence suggests that training can help reduce healthcare utilization and associated costs [28] and highlights the challenges that workforce training faces in meeting the demand for low-cost, high-quality care for increasingly diverse populations [10]. Recent findings point to the need to establish structured career pathways for HSWs that include key development areas such as data collection, care model transformation, career progression frameworks, and systems for safety and quality improvement [29]. Decision-makers in care systems face difficult economic realities, balancing public needs and aspirations with shrinking public and private resources. This necessitates challenging decisions on resource allocation to maximize performance. Reinforcing this approach, recent advocates suggest that policymakers and academic providers should redesign HSW training to meet the critical home care needs of a growing, aging population with complex needs [30].

A recent UK report on health and social care recruitment, training, and retention [31] called for a long-term, sustainable workforce strategy that emphasizes pay progression, professional development, and clear career pathways. It recommended that the Government introduce an externally validated, transferable care certificate for use across social care and the NHS, along with standardized training and progression routes to support career advancement in social care. This scoping review on optimizing CPD and HCW career pathways underlines the need for evidence on key economic issues, such as the necessity for needs assessments before launching new government-supported social programs [14]. Future research should integrate economic perspectives and address current and future needs through cost-benefit analysis (CBA) to ensure efficient resource allocation and value for money.

### Conclusion

The findings of this scoping review show there is an international scarcity of economic evidence to inform the development of career pathways for HSWs. Further research is required to build economic evidence and maximize the benefits of workforce investment costs. Expanding the home support sector could reduce costs to health systems but this requires research and evidence to inform decision-making at national and local levels.
