# Supplementary material for: A Scoping Review Investigating the International Economic Evidence to Inform the Development of a Career Pathway for Home Support Workers
Source: Public Health Rev. 2025 Mar 31;46:1607091. doi: 10.3389/phrs.2025.1607091 (PMC11995192; doi:10.3389/phrs.2025.1607091)
Supplement: Supplementary file 1 [file Table1.DOCX]

| **Concept** | **Key Terms** |
| --- | --- |
| (1a) Home Support Worker | "care worker" or “care manager” or “community care worker” or “domestic worker” or “domestic care staff” or “front-line care-worker” or “home care” or “home care agency” or “homecare agency” or “home-care agency” or “home care services” or “homecare services” or “home-care services” or “home care support worker” or “homecare support worker” or “home-care support worker” or “home carer” or “home health aide” or “home help” or “home nursing” or “home support services” or "in-home services" or "in-home care" or “paid carer” or “personal assistant” or "professional carer" or “professional home health agency staff” or “senior care worker” or “support worker” or “unlicensed assistive personnel” |
| (1b) Excluded Terms | “community support worker” or “dental devices home care” or “foster home care” or “family support worker” or “healthcare assistant” or “home care services hospital-based” or "medical trainee" or "medical residen*" or "out-of-home care" or “residency training" or "social worker” or "sexual orientation" or "veterinary" |
| (2a) Career Framework/Pathway | "advanced training" or "appraisal" or "career" or “career development” or “career framework” or “career pathway” or “career progression” or “clinical supervision” or “coaching” or “continuing professional development” or “course” or “education” or “induction” or “in-service training” or “experiential learning” or “levels of experience” or “mentorship” or “online learning” or “on-the-job training” or “orientation” or “peer-learning” or “personal skills” or “practical training” or “practice learning” or “professional development” or “professional portfolio” or “qualification” or “shadowing” or “skills development” or “specialize” or “specialism” or "supervision" or “training” or “workplace education” or “workplace learning” |
| (3) Economic measurements | “conjoint analysis” or “contingent behaviour” or “contingent valuation” or  “cost analysis” or “cost benefit” or “cost effective*” or “cost effective* analysis” or  “cost of illness” or “cost outcome” or “cost utilit*” or “cost-effectiv*” or “cost-utilit*” or “DALY” or “DCE” or “Discrete choice experiment” or “economic analys*” or “economic evaluation*” or “economic review” or “economics” or “health impact assessment” or “health related quality of life” or “impact analys*” or “Markov” or “Mental” or “opportunity cost” or “QALY” or “QoL” or “quality adjusted life year” or “return on investment” or “revealed preference” or “social cost benefit” or ”social prescribing” or “social return on investment” or “SROI” or “stated preference” or “trade-off*” or “travel cost model” |

Appendix 1

Key search terms
