# Supplementary material for: A Scoping Review Investigating the International Economic Evidence to Inform the Development of a Career Pathway for Home Support Workers
Source: Public Health Rev. 2025 Mar 31;46:1607091. doi: 10.3389/phrs.2025.1607091 (PMC11995192; doi:10.3389/phrs.2025.1607091)
Supplement: Supplementary file 2 [file Table2.DOCX]

**Inclusion and Exclusion Criteria**

| Inclusion criteria:   - - 1. Relates to ‘Home Support Workers’ or alternative terms (as defined by Key Search Terms).     2. Relates to private, commercial, publicly-funded, not-for-profit or privately arranged home support services that are paid for.     3. Relates to ‘Career Frameworks or Pathways’ or career development, or associated terms (as defined by the Key Search Terms).     4. Relates to any age group of people receiving home support (children to older adults).     5. Published in English or translated to English language.     6. Any publication date (no publication date limit applied). |
| --- |

| Exclusion criteria:   1. Refers only to community support workers, dental devices home care, foster home care, family support worker, healthcare assistant, home care services hospital-based, medical trainee, medical residents, out-of-home care, residency training, social worker, sexual orientation, or veterinary care. 2. Refers only to unpaid family or informal carers (not paid Home Support Workers). 3. Does not mention or discuss ‘Career Frameworks or Pathways’ (as defined by Key Search Terms). 4. Published prior to 2013. 5. Published in language other than English and no translation available. |
| --- |

**Grading System**

**Evidence Grades:**

I- Systematic Review

II- Research or evaluation using recognised method (Quantitative, Qualitative or Mixed method)

III- Review of literature or policy, review of evidence, submitted to a commission or to develop a white paper

IV- Case study, descriptive account

V- Commentary: comment, editorial, discussion paper, roundtable discussion

**Relevance Grades (Country):**

1. Relates to home support workers in Ireland (or worldwide)
2. Relates to a similar professional group or another country
3. Relates to a similar professional group and another country

**Theme 1- Subtheme: Cost-Benefits of Employment and Training (7 articles)**

| **Full reference of article** | **Evidence Grade** | **Relevance (Country)** | **Theme**  **Subtheme** | **Summary of Key Findings or Insights** |
| --- | --- | --- | --- | --- |
| Luz, C. C., Hanson, K. V., Hao, Y., & Spurgeon, E. (2018). Improving Patient Experiences and Outcomes Through Personal Care Aide Training. *Journal of patient experience*, *5*(1), 56–62. https://doi.org/10.1177/2374373517724349 | II- Programme evaluation | B- Effects of personal care aide training (USA) | Theme 1- Cost-Benefits of Employment and Training (cost-benefits of training) | - The rapidly aging US population is resulting in major challenges including delivering quality care at lower costs in the face of a critical health-care workforce shortage. The movement toward home care has dramatically increased the need for qualified, paid personal care aides (PCAs). Adequate PCA training that focuses on skills for person-centered, at home support is an imperative. - This study provides evidence that clients of PCAs who have completed a comprehensive, evidence based PCA training program, titled Building Training…Building Quality (BTBQ) [*TP], report higher satisfaction and better health outcomes, compared to clients of PCAs with lesser or other training. - A mixed-methods, quasi-experimental design was used to compare self-reported survey responses from clients of BTBQ-trained PCAs (treatment group) with responses from clients of non-BTBQ-trained PCAs (control group). Clients of BTBQ-trained PCAs had significantly fewer falls and emergency department visits compared to clients whose PCAs had no BTBQ training (P < .05). - The authors conclude that BTBQ-like PCA training reduces costly adverse events. |
| Luz C, Hanson K. (2015) Filling the Care Gap: Personal Home Care Worker Training Improves Job Skills, Status, and Satisfaction. Home Health Care Management & Practice. 27(4):230-237. doi:10.1177/1084822315584316 | II- Programme evaluation | B- Training programme (USA) | Theme 1- Cost-Benefits of Employment and Training (cost-benefits of training) | - With an aging population and provider shortages, personal care aides (PCAs) hold potential for providing low-cost, high-quality in-home supports and services. They comprise an unprecedented workforce in terms of size and rapid growth. However, this workforce is also characterized by costly high-turnover rates that threaten quality of care and outcomes. It is imperative that measures be taken to improve PCA skills and stabilize their employment. - In 2010, a PCA training program was developed titled “Building Training . . . Building Quality” as part of a national demonstration. - Key findings were that learners’ skills, employability, and job satisfaction significantly improved, and “intent to stay” was associated with increased confidence in ability to do the job. |
| Fong, M. C., Russell, D., Brickner, C., Gao, O., Vito, S., & McDonald, M. (2022). Medicaid long-term care workforce training intervention and value-based payment metrics. *Health services research*, *57*(2), 340–350. https://doi.org/10.1111/1475-6773.13930 | II- Research (Quant) | B- Workforce training in long-term care (USA) | Theme 1- Cost-Benefits of Employment and Training (cost-benefits of training) | - This study aimed to examine the impact of a scaled implementation of workforce training intervention on value-based payment measures in a large home-based Medicaid managed long-term care plan population in New York. - Study data was managed long-term care clients' health assessments from the Uniform Assessment System of New York merged with paid claims, home health aide operational visit data, and workforce training rosters between 2018 and early-2020. A quasi-experimental design was used. Exposure and control groups were constructed using the proportion of service hours delivered by trained aides between clients' baseline and follow-up/outcome assessments. Multivariate logistic generalized linear and additive models were estimated to examine associations between exposure to trained aides and value-based payment measures. The analytic sample consisted of 19,212 pairs of assessments from 13,320 long-term care clients continuously enrolled in the plan between baseline and follow-up/outcome assessments. Matched assessment pairs were 6-10 months apart. - Over 27% of the study population (n = 3656 clients) received services from one or more of 8683 trained aides. Statistically significant associations were observed for four of seven value-based payment measures; however, the presence and magnitudes of positive training effects differed by client service needs. With covariate adjustment, workforce training had the largest estimated positive impacts on rates of flu vaccination among average-need clients (1.60%,), not experiencing uncontrolled pain among above-average-need clients (0.69%), stable/improved pain intensity among heavy-need clients (1.25%,), and stable/improved shortness of breath among light-need clients (0.88%, SE = 0.003). - Although the study shows mixed associations between scaled workforce training implementation and value-based payment metrics, it shows that workforce training could benefit high-need long-term care recipients. Health indicators more sensitive to the daily support provided by direct care workers should be integrated into value-based health care models. |
| Kemeny, M. E., & Mabry, J. B. (2017). Making meaningful improvements to direct care worker training through informed policy: Understanding how care setting structure and culture matter. *Gerontology & geriatrics education*, *38*(3), 295–312. https://doi.org/10.1080/02701960.2015.1103652 | II- Research (Mixed method) | B- Learning and working environments in long term care (USA) | Theme 1- Cost-Benefits of Employment and Training (cost-benefits of training) | - A well-intentioned policy governing the training of direct care workers (DCWs) who serve older persons, in practice, may become merely a compliance issue for organizations rather than a meaningful way to improve quality of care. - This study investigates the relationships between best practices in DCW training and the structure and culture of long-term support service (LTSS) organizations. Using a mixed-methods approach to analyzing data from 328 licensed LTSS organizations in Pennsylvania. - The findings suggest that public policy should address methods of training, not just content, and consider organizational variations in size, training evaluation practices, DCW integration, and DCW input into care planning. Effective training also incorporates support for organizations and supervisors as key aspects of DCWs' learning and working environment. |
| Ayalon, L., & Shinan-Altman, S. (2021). Tension between reality and visions: Lessons from an evaluation of a training program of paid elder care workers. *Health & social care in the community*, *29*(6), 1915–1924. https://doi.org/10.1111/hsc.13305 | II- Programme evaluation | B- Training programme for elder care workers (Israel) | Theme 1- Cost-Benefits of Employment and Training (cost-benefits of training) | - The present study is based on a 3-year evaluation of an Israeli training program for local paid elder care workers, called, 'community care'. Interviews were conducted with all stakeholders involved in the program, including program developers, facilitators, funders, trainees, dropouts, graduates, employers and older care recipients. - Qualitative thematic analysis was used. Analysis was supplemented by quantitative data concerning the program's inputs, outputs, and outcomes. - The program had multiple strengths, including a substantial funding stream and a highly skilled and committed team. Yet, out of 130 participants, 94 completed the program and 31 worked as care workers afterwards. - Three main challenges to the efficacy of the training program were identified. A first challenge stems from the gap between the program's vision and real-life requirements and constraints. The second challenge concerns a disagreement between stakeholders concerning the definition of the new community care profession as an opportunity to empower trainees and encourage personal growth versus the community care worker as being no different from the traditional direct paid carer. A third challenge concerns the program's lack of integration between personal/physical care on the one hand and emotional and psychological care, on the other hand. The findings stress the importance of adequately conducting a needs assessment prior to embarking on a new social program and the tension between an ideal prototype and real-life constraints. The findings also stress the necessity of top-down processes, supported by the government to the development of a new profession of community elder care. |
| Bradley, P. (2015) How to do the Care Certificate Standards in 10 hours, for £36. British Journal of Healthcare Assistants, 9, 11, Health Matters. https://doi.org/10.12968/bjha.2015.9.11.530 | V- Commentary | C- Assessment of Care Certificate training (England) | Theme 1- Cost-benefits of Employment and Training (cost-Benefits of training or certification) | - Describes the process of training and attaining a Care Certificate entirely online. Argues that it is not acceptable to be assessed online and not in the work environment. The commercialisation of certification processes should not risk quality. |
| Snyder, C. R., Dahal, A., & Frogner, B. K. (2018). Occupational mobility among individuals in entry-level healthcare jobs in the USA. *Journal of advanced nursing*, *74*(7), 1628–1638. https://doi.org/10.1111/jan.13577 | II- Research (Quant) | C- Job movement from entry level healthcare occupations to personal care/services occupations (USA) | Theme 1- Cost-Benefits of Employment and Training (occupational mobility) | - The aim of this study was to explore career transitions among individuals in select entry-level healthcare occupations. Entry-level healthcare occupations are among the fastest growing occupations in the USA. Public perception is that the healthcare industry provides an opportunity for upward career mobility given the low education requirements to enter many healthcare occupations. The assumption that entry-level healthcare occupations, such as nursing assistant, lead to higher-skilled occupations, such as Registered Nurse, is under-explored. - The researchers analysed data from the Panel Study of Income Dynamics, which is a nationally representative and publicly available longitudinal survey of US households. Using longitudinal survey data, they examined the job transitions and associated characteristics among individuals in five entry-level occupations at the aide/assistant level over 10 years timeline (2003-2013) to determine whether they stayed in health care and/or moved up in occupational level over time. - This study found limited evidence of career progression in health care in that only a few of the individuals in entry-level healthcare occupations moved into occupations such as nursing that required higher education. While many individuals remained in their occupations throughout the study period, the results show that 28% of the sample moved out of these entry-level occupations and into another occupation. The most common "other" occupation categories were "office/administrative" and "personal care/services occupations." Whether these moves helped individuals advance their careers remains unclear. - The authors argue that employers and educational institutions should consider efforts to help clarify pathways to advance the careers of individuals in entry-level healthcare occupations. |

**Theme 2- Subtheme:** **Organisational Economic Perspectives (3 articles)**

| **Full reference of article** | **Evidence Grade** | **Relevance (Country)** | **Theme**  **Subtheme** | **Summary of Key Findings or Insights** |
| --- | --- | --- | --- | --- |
| Julin, A. (2020). “We don’t have a choice–we need to change!” A case study on experienced efficiency and the manager role in the home care. Thesis. Lund University. Dept Political Science. https://lup.lub.lu.se/luur/download?func=downloadFile&recordOId=9009734&fileOId=9016272 | II- Research (Qual) | Care manager efficiency role and behaviours (Sweden) | Theme 2- Organisational Economic Perspectives (efficiency of organisations and care manager’s role and behaviours) | - Efficiency requirements are today found in all Swedish municipalities and regions. With the care manager in lead, the home care organisations are today expected to find efficient solutions to organisational problems. The case of the care manager is studied through a thematic analysis of interview data and recruitment announcements. - From 11 interviews with care managers and 23 care managers recruitment profiles, certain similarities and differences are found. By applying theories such as transformational leader and manager role as well as leadership behaviours on the collected material, this thesis aims at broadening knowledge on the relations between the efficiency and the manager’s role, including their tasks and the organisation of home care. - This thesis shows that the care managers are well aware of the efficiency requirements in the elderly care, however, in contrast to earlier research, they do not perceive the political decisions as demanding as their fellow managers did. Furthermore, the role of the care manager is a complex position which requires different behaviours. Consequently, the managers see a new form of home care appearing, where the main tasks of the managers are to guide the employees towards a smarter use of the resources. This shows a shift in thinking and which will have to follow in the rest of the society, too. |
| Kessler, I., Steils, N., Esser, A., & Grant, D. (2021). Understanding career development and progression from a healthcare support worker perspective. Part 1/2. British Journal of Healthcare Assistants, 15(11), 526-531. | V- Commentary | B- Career progression and development in healthcare support worker roles (England) | Theme 2- Organisational Economic Perspectives (organisational capacity to implement and fulfil training agendas) | - With access to and progression within the HCSW role seen as a means of opening-up the NHS workforce to diverse socio-economic groups often facing deep-seated labour market challenges (HEE, 2014b). More prosaically, career pathways for HCSWs into pre-registration nurse training, possibly via the nursing associate role, have been seen as a grow-your-own way of helping to address shortages in registered nurses. Alongside this is the pre-eminent objective of HCSW career development: supporting HCSWs to grow within the role itself, so enabling them to deliver high-quality care. - Such a crowded HCSW learning and development agenda generates a host of questions. These questions centre on the organisational balance to be struck in pursuing the myriad HCSW development aims outlined above, not least within the context of a training activity which extends beyond support workers to the wider NHS workforce. - Indeed, in this broader context, issues of organisational capacity to deliver on the HCSW agenda arise, alongside concerns about how to align the pursuit of different aims with the appropriate training and development programmes. For example, alongside HCSW apprenticeships designed to support progression through different job roles, other programme options are available, such as the higher development award, that is suited to HCSWs in role, who wish to undertake continuing professional development while staying in their existing role. |
| Kessler, I., Steils, N., Esser, A., & Grant, D. (2022). Understanding career development and progression from a healthcare support worker perspective. Part 2. British Journal of Healthcare Assistants, 16(1), 6-10. | V- Commentary | B- Career progression and development in healthcare support worker roles (England) | Theme 2- Organisational Economic Perspectives (organisational capacity to implement and fulfil training agendas) | - The article presents perspective and discussion on the importance of functional skills to career progression. Topics include absence of organisational funding for HCSW training contrasted with the funding of HCSWs; and feeling the COVID-19 situation greatly affecting the training opportunities because of staff shortages. |

**Theme 3- Subtheme:** **Service Economic Perspectives (2 articles)**

| **Full reference of article** | **Evidence Grade** | **Relevance (Country)** | **Theme**  **Subtheme** | **Summary of Key Findings or Insights** |
| --- | --- | --- | --- | --- |
| McGilton, K. S., Rochon, E., Sidani, S., Shaw, A., Ben-David, B. M., Saragosa, M., Boscart, V. M., Wilson, R., Galimidi-Epstein, K. K., & Pichora-Fuller, M. K. (2017). Can We Help Care Providers Communicate More Effectively With Persons Having Dementia Living in Long-Term Care Homes? *American journal of Alzheimer's disease and other dementias*, *32*(1), 41–50. https://doi.org/10.1177/1533317516680899 | II- Programme evaluation | B- Dementia training in long term care (Canada) | Theme 3- Service Economic Perspectives (costs and benefits of workforce training in long-term care) | - Effective communication between residents with dementia and care providers in long-term care homes (LTCHs) is essential to resident-centered care. - To determine the effects of a communication intervention on residents' quality of life (QOL) and care, as well as care providers' perceived knowledge, mood, and burden. - The intervention included (1) individualized communication plans, (2) a dementia care workshop, and (3) a care provider support system. Pre- and postintervention scores were compared to evaluate the effects of the intervention. A total of 12 residents and 20 care providers in an LTCH participated in the feasibility study. - The rate of care providers' adherence to the communication plans was 91%. Postintervention, residents experienced a significant increase in overall QOL. Care providers had significant improvement in mood and perceived reduced burden. The results suggest that the communication intervention demonstrates preliminary evidence of positive effects on residents' QOL and care providers' mood and burden. |
| Franzosa, E., Tsui, E. K., & Baron, S. (2018). Home Health Aides' Perceptions of Quality Care: Goals, Challenges, and Implications for a Rapidly Changing Industry. *New solutions : a journal of environmental and occupational health policy : NS*, *27*(4), 629–647. https://doi.org/10.1177/1048291117740818 | II- Research (Qual) | B- Home care aides views on quality care (USA) | Theme 3- Service Economic Perspectives (costs and benefits of payment models) | - Home care payment models, quality measures, and care plans are based on physical tasks workers perform, ignoring relational care that supports clients' cognitive, emotional, and social well-being. As states seek to rein in costs and improve the efficiency and quality of care, they will need to consider how to measure and support relational care. - Four focus groups (n = 27) of unionized, agency-based New York City home health aides. - Workers reported aide-client relationships were a cornerstone of high-quality care, and building them required communication, respect, and going the extra mile. Since much of this care was invisible outside the worker-client relationship, aides received little supervisory support and felt excluded from the formal care team. - Aligning payment models with quality requires understanding the full scope of services aides provide and a quality work environment that offers support and supervision, engages aides in patient care, and gives them a voice in policy decisions. |

- **Theme 4- Subtheme:** **Sector Economic Perspectives (5 articles)**

| **Full reference of article** | **Evidence Grade** | **Relevance (Country)** | **Theme**  **Subtheme** | **Summary of Key Findings or Insights** |
| --- | --- | --- | --- | --- |
| Ramos LR, d’Orsi E and Simões EJ (2023) Editorial: Longevity with functionality and quality of life. Front. Aging 4:1281737 <https://doi.org/10.3389/fragi.2023.1281737> | V- Editorial | B- Home care for older people (worldwide) | Theme 4- Sector Economic Perspective (economic evaluation methods) | - The purpose of this paper is to was to gather evidence on risk factors for loss of functional capacity and effective interventions to preserve or improve functional capacity of the elderly population - Health promoting interventions that prevents functional loss both in physical and mental terms thus promoting quality of life (QOL). - Highlights the socioeconomic and health implications of the longevity revolution and asserts that successful ageing in this new paradigm should include health promoting interventions that prevent functional loss and promote quality of life. This view supports the argument for economic evaluations of home support interventions in the context of rising healthcare costs. |
| Velazquez, F., Fox, S., & Chatha, D. (2022). Adaptive Strategies to Building a Sustainable Workforce: Health Care Support Worker (HCSW) and the Health Career Access Program (HCAP). International Journal of Integrated Care (IJIC), 22. | II- Programme evaluation | B- Large-scale career building strategy for health care careers (Canada) | Theme 4- Sector Economic Perspectives (cost-benefit of large-scale policy-directives or workforce development interventions) | - The COVID-19 pandemic has had a tremendous impact on organizations. Health care in particular has been affected in many ways. Health care professionals have been working around the clock to support patients and families during these tumultuous times. It is well known that these demands have taken a significant toll on staff which has led to burnout. To ensure that the health care workforce remains strong, the British Columbia Government has implemented the Health Career Access Program (HCAP). Fraser Health coordinates the management of HCAP in the region that it serves (1.8 million people, 1 out of 3 British Columbians). - The British Columbia Government opted to introduce a new career building strategy in the midst of a pandemic. Through HCAP, individuals have the opportunity to apply to become a Health Care Support Worker (HCSW). The HCSW role is an entry-level position that provides non-direct care to residents in AL and LTC. While working, HCSWs are afforded the opportunity to go to school to become health care assistants, with all costs covered by the Government, including their salary. The Return of Service agreement ensures that staff remain and there is stability and sustainment in the workforce. - Using key change management principles, the Fraser Health HCAP Project Team has been supporting the HCSW, the care team, as well as residents and families. Focused on building a team-based care environment the project team creates opportunities for open dialogue and engagement. This supports building "Awareness" and "Desire." Preparing resources such as the "HCSW Can Do-Can Not Do" list in addition to the shadow-shifts and buddy-pairing amongst other tactics, allow for the "Knowledge" and "Ability" to show up. By investing in, and supporting the growth of an individual, there is emphasis on sustainment and longevity (Reinforcement). - Although early in the implementation phase, the feedback has been positive from both the provider and resident-experience. A Logic Model is being built to measure the true impact of the program, and how the model can inform the future of the provision of AL and LTC services. - While working in health care this past year has certainly brought its challenges, there have also been opportunities. The broad interest in HCAP has shown that rather than shying away from health care, the opposite appears to be true in that people want to be part of the field. - While having taken a system-wide engagement approach, HCAP focuses on current and immediate future needs. It is highly dependent on the supplementary funding from the government and questions will remain as to how long funding will be available. |
| Community Integrated Care (2021) Unfair to care: understanding the social care pay gap and how to close it: full report  pp. 64. Epsom, UK. https://www.unfairtocare.co.uk/wp-content/uploads/2021/07/Unfair-To-Care-Full-Report-Single-Pages.pdf | II- Research (Mixed method) | B- Costs and benefits of social care workforce (England) | Theme 4- Sector economic perspectives (funding/resourcing rising home care costs, why and how to pay for home care) | - A report on the social care workforce crisis, providing in-depth evidence that frontline carers receive an unjust deal in comparison to other public funded industries and challenging the stereotype that social care is a ‘low-skilled’ sector. This research indicates many social care workers would be paid up to 39% more – an additional £7,000 – if they worked in other public funded sectors. With the role matching or exceeding the level of skill and accountability as professions such as healthcare assistants, police community support officers, and senior teaching assistants, the roots of the workforce crisis are laid bare. - The report also reveals that the sector loses 34% of its workforce every year – the issue of low pay is at the heart of the crisis affecting social care; social care contributes £46bn to the national economy annually; every year, social care creates 1.65m jobs, which each contribute to their local economy. The report calls on the Government to: provide an immediate and fair pay rise to all frontline social care workers, as an initial step towards achieving equity with the public sector and NHS; immediately instruct an expert-led social care workforce review, which has cross-party support – this should deliver a system of fair and objective pay benchmarks and bandings for all roles, to deliver parity with the NHS and other publicly-funded sectors at the earliest opportunity; create a wider strategy to make social care a viable, respected, and sustainable career – this includes a greater focus on learning and development and qualifications, registration, the development of career pathways and improving the image of the sector. |
| Lu, J. (2015) Status Quo, Problems and Countermeasure Research of Home-Based Care Service Market in China—A Case Study of Sichuan Province. Open Journal of Social Sciences, 3, 150-156. doi: 10.4236/jss.2015.311020. | II- Research (Qual) | C- Supply and demand challenges in home care (China) | Theme 4- Sector Economic Perspectives (service market, business models and profit margins) | - This paper makes a case study of Sichuan Province where a large aging population lives. Using social investigation, 350 aged community people were interviewed to discuss the conflict between supply and demand in the current home-based care service market and the problems arising out of the market-oriented operation. In addition, this paper brings about specific counter measures and recommendations, with a view to give full play to the role of market- oriented mechanism in optimized allocation of resources, and to inspire participance of various forces into home-based care service market, thus diversifying the home-based care service providers and provision modes in China, and greatly improving the QOL (quality of life) of the elderly. |
| Rooijackers, T. H., Metzelthin, S. F., van Rossum, E., Kempen, G. I. J. M., Evers, S. M. A. A., Gabrio, A., & Zijlstra, G. A. R. (2021). Economic Evaluation of a Reablement Training Program for Homecare Staff Targeting Sedentary Behavior in Community-Dwelling Older Adults Compared to Usual Care: A Cluster Randomized Controlled Trial. *Clinical interventions in aging*, *16*, 2095–2109. https://doi.org/10.2147/CIA.S341221 | II- Research (Quant) | B- Cost-effectiveness and cost-utility of staff reablement training (Holland) | Theme 4- Sector Economic Perspectives (the need for better economic evaluation methods linked to outcomes) | - Training and supporting homecare staff in reablement aims to change staff behavior from "doing for" to "doing with" older adults and is assumed to benefit the health and quality of life of older adults and reduce healthcare utilization and costs. This study evaluated the cost-effectiveness and cost-utility of the staff reablement training program "Stay Active at Home" (SAaH) from a societal perspective. - An economic evaluation was embedded in a 12-month cluster randomized controlled trial. Ten Dutch homecare nursing teams participated (n = 313 staff members), of which five teams were trained in reablement and the other five provided usual care. Cost and effect data were collected from 264 older adults at baseline, 6 and 12 months. Costs included "intervention," "healthcare," and "patient and family" costs (collectively, societal costs) and were assessed using questionnaires and client records or estimated by bottom-up micro-costing. Effects included sedentary behavior and quality-adjusted life years (QALYs). Multiple imputed bootstrapped data were used to generate cost-effectiveness planes and acceptability curves. - No statistically significant differences were observed between the intervention and control group in terms of sedentary time (adjusted mean difference: 4.8 minutes [95% CI -26.4, 36.0]), QALYs ( 0.01 [95% CI -0.03, 0.04]), and societal costs ( €2216 [95% CI -459, 4895]), except lower costs for domestic help in the intervention group ( €-173 [95% CI -299, -50]). The probability that SAaH was cost-effective compared to usual care ranged from 7.1% to 19.9%, depending on the willingness-to-pay (WTP) (€0‒€50,000)/minute of sedentary time averted and was 5.9% at a WTP of €20,000/QALY gained. - SAaH did not improve outcomes or reduce costs and was not cost-effective from a societal perspective compared to usual care in Dutch older adults receiving homecare. Consequently, there is insufficient evidence to justify widespread implementation of the training program in its current form. |
